# Melanophilin Stimulates Myosin-5a Motor Function by Allosterically Inhibiting the Interaction between the Head and Tail of Myosin-5a

**DOI:** 10.1038/srep10874

**Published:** 2015-06-03

**Authors:** Lin-Lin Yao, Qing-Juan Cao, Hai-Man Zhang, Jie Zhang, Yang Cao, Xiang-dong Li

**Affiliations:** 1 Group of Cell Motility and Muscle Contraction, State Key Laboratory of Integrated Management of Insect Pests and Rodents, Institute of Zoology, Chinese Academy of Sciences, Beijing 100101, China; 2 University of Chinese Academy of Sciences, Beijing 100049, China

## Abstract

The tail-inhibition model is generally accepted for the regulation of myosin-5a motor function. Inhibited myosin-5a is in a folded conformation in which its globular tail domain (GTD) interacts with its head and inhibits its motor function, and high Ca^2+^ or cargo binding may reduce the interaction between the GTD and the head of myosin-5a, thus activating motor activity. Although it is well established that myosin-5a motor function is regulated by Ca^2+^, little is known about the effects of cargo binding. We previously reported that melanophilin (Mlph), a myosin-5a cargo-binding protein, is capable of activating myosin-5a motor function. Here, we report that Mlph-GTBDP, a 26 amino-acid-long peptide of Mlph, is sufficient for activating myosin-5a motor function. We demonstrate that Mlph-GTBDP abolishes the interaction between the head and GTD of myosin-5a, thereby inducing a folded-to-extended conformation transition for myosin-5a and activating its motor function. Mutagenesis of the GTD shows that the GTD uses two distinct, non-overlapping regions to interact with Mlph-GTBDP and the head of myosin-5a. We propose that the GTD is an allosteric protein and that Mlph allosterically inhibits the interaction between the GTD and head of myosin-5a, thereby activating myosin-5a motor function.

Class V myosin (Myo5) is one of the oldest classes of myosins, which is distributed from lower eukaryotes, such as yeast, to vertebrate cells^1^. Thus far, the most well-characterized Myo5 is vertebrate Myo5a, which is a processive motor that is capable of individually moving along an actin filament for several steps without dissociation^2^,^3^,^4^,^5^,^6^. Myo5a contains a motor domain and an extended lever arm followed by a coiled-coil dimerizing region and a C-terminal globular tail domain (GTD)^7^.

Myo5a is responsible for the transportation and localization of a number of vesicles, including melanosomes in melanocytes (for a review, see^8^). Melanosomes associate with Myo5a via Rab27a and melanophilin (Mlph)^9^,^10^,^11^. Rab27a localizes to melanosome membranes and interacts with Mlph. Mlph contains an N-terminal Rab27a-binding domain and two independent Myo5a-binding regions^9^,^10^. The first interaction occurs between the melanocyte-specific exon-F in the Myo5a tail and Mlph-EFBD (Exon-F Binding Domain; residues 241–400), and the second interaction occurs between the GTD of Myo5a and Mlph-GTBD (Globular Tail domain-Binding Domain; residues 147–240)^12^. Spudich and colleagues narrowed down Mlph-GTBD to a 26-residue peptide (residues 176–201)^13^. Cell biology studies have demonstrated that both the interaction between exon-F and Mlph-EFBD and the interaction between the GTD and Mlph-GTBD are essential for rescuing the melanosome transport defect in *dilute* and *leaden* melanocytes^9^,^14^.

A critical question is how the motor function of Myo5a is regulated. A tail-inhibition model for Myo5a regulation is generally accepted. In this model, Myo5a in the inhibited state is in a folded conformation such that its tail interacts with its head and inhibits motor activity, and high Ca^2+^ or cargo binding may reduce the interaction between the head and tail, thus activating motor activity^15^,^16^,^17^. Ca^2+^ activation of Myo5a’s motor function has been the subject of intense investigation^15^,^16^,^17^,^18^,^19^,^20^,^21^,^22^,^23^. Ca^2+^-induced activation of Myo5a’s ATPase activity is accompanied by a folded-to-extended conformation transition^16^. Truncation analyses of Myo5a have indicated that Myo5a motor function is inhibited by the GTD, and this inhibition is abolished by Ca^2+^^19^,^20^. We recently found that the calmodulin (CaM) in the first IQ motif participates in the interaction between the head and the GTD and is responsible for the activation of Myo5a by Ca^2+^^24^. Thus, it is likely that Ca^2+^ induces a conformational change in the CaM in IQ1, thereby preventing an interaction between the head and the GTD and causing motor function activation.

However, little is known about the effects of cargo binding on the motor function of Myo5a. Because the tail of Myo5a not only functions as a cargo binding site but also serves as a key regulatory component of Myo5a, Sellers and colleagues proposed that the binding of cargo to the tail might activate the motor activity of Myo5a^15^. Consistent with this prediction, we found that Mlph directly stimulates the actin-activated ATPase activity of Myo5a^25^. Recently, Trybus and colleagues demonstrated at the single-molecule level that Mlph significantly increases the number of processively moving Myo5a molecules^26^. However, it is not clear whether Mlph activates the Myo5a motor by the same mechanism as Ca^2+^; i.e., by abolishing the tail inhibition of the head.

In this study, we found that Mlph-GTBDP, the 26-residue Myo5a-GTD binding peptide of Mlph identified by Spudich and colleagues^13^, is capable of activating the motor function of Myo5a. We demonstrate that Mlph-GTBDP abolishes the interaction between the GTD and the head of Myo5a, thus inducing a folded-to-extended conformational transition of Myo5a and activating its motor function. Mutagenesis of the GTD demonstrated that the GTD uses distinct regions to interact with Mlph-GTBDP and the head of Myo5a. We therefore propose that the GTD of Myo5a is an allosteric protein and that Mlph-GTBDP binding allosterically inhibits the interaction between the GTD and the head of Myo5a, thus activating the head’s motor function.

## Results

### Mlph-GTBDP stimulates the ATPase activity of Myo5a

**Figure 1:**
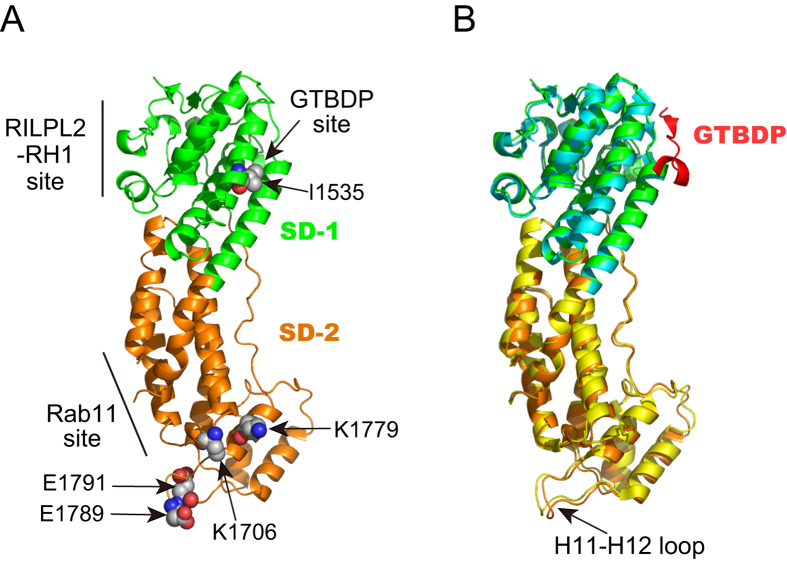
Structural comparison of Myo5a-GTD and Myo5a-GTD/Mlph-GTBDP. (**A**) Ribbon representation of the human Myo5a-GTD structure (PDB ID: 4LX1) showing binding sites for Mlph-GTBDP, RILPL2-RH1, and Rab11. The residues mutated in our study are shown as spheres. (**B**) Overlap of the crystal structures of the human Myo5a-GTD (PDB ID: 4LX1) and Myo5a-GTD/Mlph-GTBDP complex (PDB ID: 4LX2). The overlap reveals a relatively large conformation change in the H11-H12 loop upon Mlph-GTBDP binding. Subdomain-1 (SD-1) and subdomain-2 (SD-2) are shown in green and orange for the apo-GTD and cyan and yellow for the GTD/Mlph-GTBDP complex.

**Figure 2:**
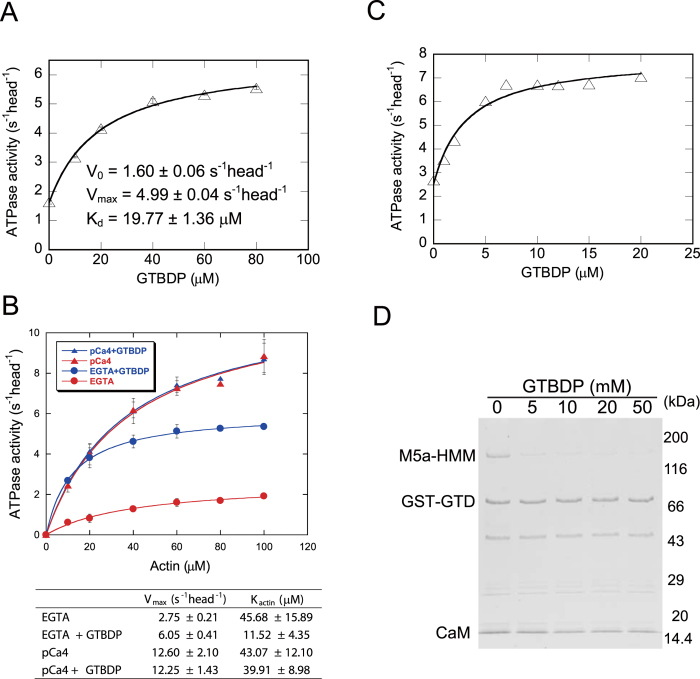
Mlph-GTBDP stimulates the ATPase activity of Myo5a by inhibiting the interaction between the head and GTD. (**A**) Mlph-GTBDP stimulates the ATPase activity of Myo5a-FL. Stimulation of the ATPase activity of Myo5a-FL by Mlph-GTBDP was fit to a hyperbolic equation: V = V_0_ + V_max_ [Mlph-GTBDP]/(Kd + [Mlph-GTBDP]), where V_0_ is the activity in the absence of Mlph-GTBDP, V_max_ is the maximal activity in the presence of saturated Mlph-GTBDP, and Kd is the apparent dissociation constant between Mlph-GTBDP and Myo5a. The ATPase assays were conducted under EGTA conditions and in the presence of 40 μM actin. Values are the average ± error for two independent assays. (**B**) Actin dependence of the ATPase activity of Myo5a-FL in the absence or presence of Mlph-GTBDP. ATPase assays under EGTA or pCa4 conditions were conducted in the absence or presence of 60 μM Mlph-GTBDP. Values are the average ± std of three independent assays. (**C**) Mlph-GTBDP attenuates the GST-GTD-mediated inhibition of Myo5a-HMM ATPase activity. ATPase assays were conducted in the presence of 40 μM actin and 0.5 μM GST-GTD. (**D**) Mlph-GTBDP inhibits the interaction between Myo5a-HMM and the GTD. GST pull-down of GST-GTD with Myo5a-HMM was performed in the presence of the indicated concentration of Mlph-GTBDP. The resulting samples were analyzed by SDS-PAGE and visualized by CBB staining.

Of the two Myo5a-binding sites Mlph-GTBD and Mlph-EFBD, we previously demonstrated that Mlph-GTBD is capable of stimulating the actin-activated ATPase activity (hereafter referred to as ATPase activity) of Myo5a^25^. Recently, structural studies^27^,^28^ have shown that Mlph-GTBDP, a 26-residue Myo5a-GTD binding peptide within Mlph-GTBD^13^, binds to a cleft in subdomain-1 (SD-1) of Myo5a-GTD (Fig. 1). Here, we examined whether Mlph-GTBDP is capable of stimulating the ATPase activity of Myo5a. As shown in Fig. 2A, Mlph-GTBDP significantly stimulated the ATPase activity of Myo5a-FL (full-length Myo5a) under EGTA conditions. The kinetics of Mlph-GTBDP-mediated stimulation follows the Michaelis-Menten equation with a V_max_ of 4.99 ± 0.04 s^−1^ head^−1^ and a K_d_ of 19.77 ± 1.36 μM for Mlph-GTBDP.

To further characterize the activation of the Myo5a-FL ATPase activity by Mlph-GTBDP, we compared the actin dependence of the Myo5a-FL ATPase activity in the absence and presence of 60 μM Mlph-GTBDP. Under EGTA conditions, 60 μM Mlph-GTBDP increased the V_max_ of the Myo5a-FL ATPase activity from 2.75 ± 0.21 to 6.05 ± 0.41 s^−1^ head^−1^ and decreased the K_actin_ from 45.68 ± 15.89 to 11.52 ± 4.35 μM (Fig. 2B). By contrast, 60 μM Mlph-GTBDP did not significantly affect the ATPase activity of Myo5a-FL under pCa4 conditions (Fig. 2B). The V_max_ and the K_actin_ of Myo5a-FL were ~12 s^−1^ head^−1^ and ~40 μM, respectively, regardless of Mlph-GTBDP. The V_max_ under EGTA conditions in the presence of 60 μM Mlph-GTBDP was approximately 50% of that under pCa4 conditions. Based on the K_d_ of Mlph-GTBDP association with Myo5a-FL (19.77 μM), we calculated that only ~75% of Myo5a-FL was activated by 60 μM Mlph-GTBDP (equal to 60/(60 + 19.77)). Thus, we expected that the ATPase activity of Myo5a-FL under EGTA conditions in the presence of saturating Mlph-GTBDP was approximately 66.7% (equals to 50%/75%) of that in pCa4 conditions.

We previously characterized the ATPase activity of two Myo5a-FL mutants, D136A and K1706A/K1779A^21^. Both mutations completely abolish inhibition of Myo5a motor function by the GTD. The ATPase activity of these two mutants under EGTA conditions was 60–70% of that under pCa4 conditions (Fig. 2 and SI Table 1 in reference^21^). These numbers are close to the ratio of Myo5a-FL ATPase activity under EGTA conditions in the presence of saturating Mlph-GTBDP versus that under pCa4 conditions, indicating that Mlph-GTBDP is capable of completely abolishing the inhibition by the GTD.

### Mlph-GTBDP induces an extended conformation of Myo5a by inhibiting the interaction between the GTD and head of Myo5a

It is well established that the tail of Myo5a, specifically the GTD, is the inhibitory domain and the inhibited state of Myo5a is formed by the binding of the GTD to the head of Myo5a^19^,^20^. Ca^2+^-induced Myo5a activation is accompanied by a conformational transition of Myo5a; i.e., from ~14 S of the folded conformation to ~11 S of the extended conformation^15^,^16^,^17^. Because Mlph-GTBDP stimulates the ATPase activity of Myo5a, we expected that, similar to Ca^2+^, Mlph-GTBDP would also induce a folded-to-extended conformation transition for Myo5a. To test this possibility, we used two approaches to examine the effects of Mlph-GTBDP on the conformation of Myo5a.

**Figure 3:**
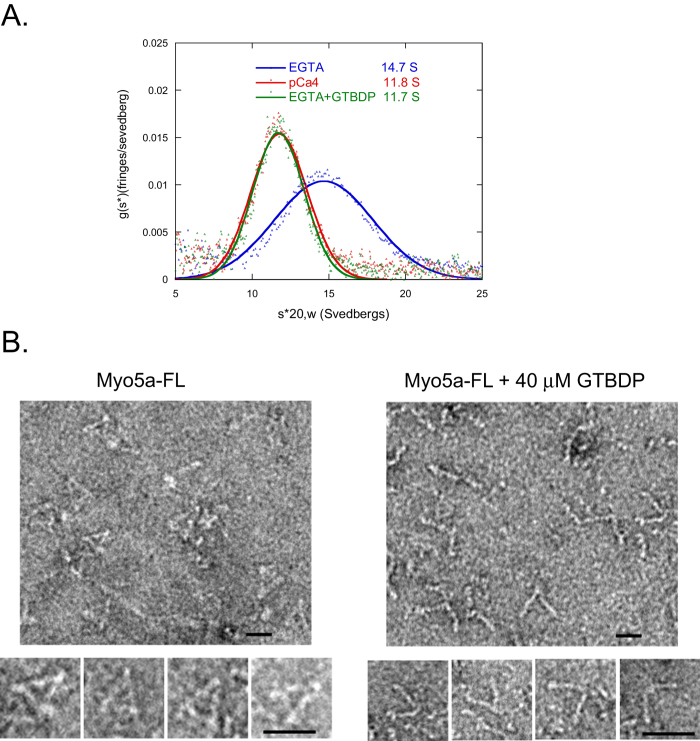
Mlph-GTBDP induces an extended conformation of Myo5a. (**A**) Analytical ultracentrifugation analysis of Myo5a-FL under EGTA conditions in the absence or presence of 40 μM Mlph-GTBDP and under pCa4 conditions. (**B**) Negative-staining electron microscopy of Myo5a-FL in the absence and presence of 40 μM Mlph-GTBDP. Bar, 25 nm.

First, we measured the sedimentation coefficient of Myo5a in the absence and presence of Mlph-GTBDP using analytical ultracentrifugation analysis. The sedimentation coefficient of Myo5a under EGTA conditions decreased from 14.7 S in the absence of Mlph-GTBDP to 11.7 S in the presence of 40 μM Mlph-GTBDP, a value that is similar to that obtained under pCa4 conditions (Fig. 3A). These results indicate that Myo5a is in an extended conformation in the presence of Mlph-GTBDP.

Second, we used negative-staining electron microscopy to visualize the conformation of Myo5a in the presence of Mlph-GTBDP. Consistent with our analytical ultracentrifugation analysis results, most Myo5a molecules were in the folded conformation in the absence of Mlph-GTBDP, and in the presence of 40 μM Mlph-GTBDP, the majority of Myo5a molecules were in the extended conformation (Fig. 3B). Note that the density of Myo5a-FL molecules in the presence of Mlph-GTBDP was significantly higher than that in the absence of Mlph-GTBDP. For clarity, we intentionally selected a less crowded field (right panel, Fig. 3B) to show the open conformation of individual Myo5a-FL molecules, which may indicate that Myo5a-FL molecules in the extended conformation attach more easily to the mica surface than those in the folded conformation.

The results described above suggest that Mlph-GTBDP stimulates the ATPase activity of Myo5a by inhibiting the interaction between the head and the GTD, thus inducing an extended conformation for Myo5a and attenuating the inhibition of the head by the GTD. Consistent with this notion, we found that Mlph-GTBDP inhibited the interaction between GST-GTD and Myo5a-HMM (Fig. 2D) and attenuated the inhibition of the Myo5a-HMM ATPase activity by GST-GTD (Fig. 2C).

### The GTD uses two distinct regions to interact with Mlph-GTBDP and the head of Myo5a

There are at least two possible mechanisms by which Mlph-GTBDP inhibits the interaction between the GTD and the head of Myo5a, i.e. Mlph-GTBDP sterically blocks the head-binding site in the GTD or Mlph-GTBDP allosterically inhibits the interaction between the GTD and the head of Myo5a. To distinguish between these possibilities, we performed a mutagenesis analysis of the Mlph-GTBDP binding site and the head binding site in the GTD.

**Figure 4:**
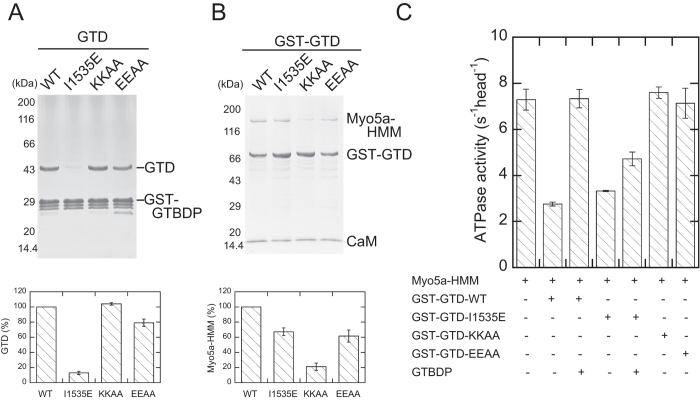
The GTD uses distinct regions to interact with Mlph-GTBDP and the head of Myo5a. (**A**) The GST pull-down of GST-Mlph-GTBDP with GTD. (**B**) The GST pull-down of GST-GTD with Myo5a-HMM. The amount of pull-downed GTD (**A**) or Myo5a-HMM (**B**) was determined by scanning densitometry (NIH imageJ program) and normalized with the density of GST-GTBDP (**A**) or GST-GTD (**B**). The values (as percentage of WT) shown are means +/− SD from three independent experiments. (**C**) Inhibition of the Myo5a-HMM ATPase activity by GST-GTD with or without Mlph-GTBDP. The ATPase assays were conducted in the presence of 40 μM actin, 0 or 0.5 μM GST-GTD, and 0 or 20 μM Mlph-GTBDP. The values shown are means +/− SD from three independent experiments. KKAA, K1706A/K1779A; EEAA, E1789A/E1791A.

The crystal structures of the Myo5a-GTD/Mlph-GTBDP complex show that Mlph-GTBDP binds to a cleft in subdomain-1 (SD-1) of the GTD, which is formed by the H3 and H5 helices and a loop preceding the H5 helix (Fig. 1). It was reported that an I1535E mutation in this cleft greatly decreases the binding affinity to Mlph-GTBDP^27^. Consistent with previous findings, we found that I1535E greatly reduced the amount of GTD pull-downed with GST-Mlph-GTBDP (Fig. 4A). By contrast, I1535E did not significantly affect the inhibition of the Myo5a-HMM ATPase activity by GST-GTD (Figure S1) and only slightly weakened the interaction between Myo5a-HMM and GST-GTD (Fig. 4B). Moreover, I1535E substantially dampened the Mlph-GTBDP-induced activation of Myo5a-HMM in the presence of GST-GTD (Fig. 4C).

The precise interaction between the head and GTD is unknown because a high resolution structure of folded, inhibited Myo5a is not available. Several studies have suggested that the head-binding site of the GTD is located in subdomain-2 (SD-2) (Fig. 1A)^21^,^27^,^28^,^29^. We previously found that two conserved basic residues, K1706 and K1779, in the SD-2 are essential for inhibiting Myo5a motor function by the GTD^21^. We found here that K1706A/K1779A mutations not only abolish the inhibition of Myo5a-HMM ATPase activity by GST-GTD (Figure S1 and Fig. 4C), but they also strongly weaken the interaction between GST-GTD and Myo5a-HMM (Fig. 4B). By contrast, K1706A/K1779A mutations did not affect the interaction between the GTD and Mlph-GTBDP (Fig. 4A).

In our previously proposed model for the interaction between the head and the GTD, the SD-2 of the GTD docked with a pocket of the head formed by the N-terminal domain, converter, and CaM bound to IQ1^21^. Recently, Velvarska and Niessing refined our model using a recently resolved crystal structure of human Myo5a-GTD^30^. In their model, the position of the H11-H12 loop is close enough to allow for potential contact with the converter or CaM bound to IQ1 (Fig. 1). To examine the role of the H11-H12 loop and the interaction between the GTD and head of Myo5a or Mlph-GTBDP, we mutated two conserved acidic residues in the H11-H12 loop, E1789 and E1791, to alanine. Similar to K1706A/K1779A mutations, E1789A/E1791A mutations strongly weakened the inhibition of Myo5a-HMM ATPase activity by GST-GTD (Figure S1 and Fig. 4C). However, unlike the GTD-K1706A/K1779A mutants, GTD-E1789A/E1791A mutants maintained substantial interaction with Myo5a-HMM (Fig. 4B). These results indicate that E1789 and E1791 are critical for inhibiting Myo5a-HMM motor function, but they are not essential for interacting with the head. By contrast, similar to K1706A/K1779A mutations, E1789A/E1791A mutations did not greatly affect the interaction between the GTD and Mlph-GTBDP (Fig. 4A), which is consistent with the structure of the GTD/Mlph-GTBDP complex that demonstrated the Mlph-GTBDP binding site in the SD-1.

Taken together, the above results indicate that the GTD uses two distinct regions to interact with the head of Myo5a and Mlph-GTBDP. Given the geometry of the GTD and the size of Mlph-GTBDP, it is unlikely that Mlph-GTBDP sterically blocks the interaction between the GTD and the head of Myo5a. Instead, these results suggest that Mlph-GTBDP allosterically inhibits the interaction between the GTD and the head of Myo5a, thus stimulating the ATPase activity of Myo5a.

## Discussion

There are two major findings in this study. First, we demonstrated that the binding of Mlph-GTBDP to the GTD inhibits the interaction between the GTD and head of Myo5a, thus disrupting the inhibition on the motor function in the head of Myo5a. This finding provides direct evidence that cargo activation of the Myo5 motor function is accompanied by a folded-to-extended conformational change in Myo5a. Second, we found that the GTD uses two distinct, non-overlapping sites to interact with Mlph-GTBDP and the head of Myo5a. Because these two binding sites are located in different subdomains of the GTD, we propose that Mlph-GTBDP allosterically inhibits the interaction between the GTD and the head of Myo5a, thus stimulating the ATPase activity of Myo5a.

In principle, all globular proteins are allosteric^31^. We propose that the GTD of Myo5a is an allosteric protein. The GTD uses two distinct, non-overlapping regions to interact with Mlph-GTBDP and the head of Myo5a. The binding site for Mlph-GTBDP in the GTD is located in a cleft in the SD-1, which is formed by the H3 and H5 helices and a loop preceding the H5 helix^27^,^28^. Upon Mlph-GTBDP binding, the GTD undergoes local conformational rearrangements in the Mlph-GTBDP binding site, enabling the hydrophobic cleft between H3 and H5 helices to accommodate Mlph-GTBDP^28^. The overall conformation of GTD alone or in complex with GTBDP is similar, with the exception of a substantial conformational change in the H11-H12 loop (Fig. 1B)^27^,^28^. However, the conformational change in the H11-H12 loop is likely due to crystal packing because the H11-H12 loop is involved in crystal packing in the apo-GTD structure, whereas the same loop is not restrained in the Mlph-GTBDP bound structure by crystal packing. These considerations suggest that Mlph-GTBDP binding does not induce a major conformational change in the GTD.

Several studies have suggested that the head-binding site in the GTD is located in the SD-2. Sequence alignment of all known Myo5 homologs revealed two large conserved surface patches in the SD-2^29^. Weisman and colleagues reasoned that the interaction between the head and GTD is an ancient, common property of Myo5 and proposed that either of the two conserved patches in the SD-2 may be the head-binding site^29^. Consistent with this hypothesis, we previously demonstrated that two highly conserved basic residues (K1706 and K1779) located on the conserved surface patch of SD-2 are essential for autoinhibition^21^. In this study, we identified two conserved acidic residues (E1789 and E1791) in the H11-H12 loop to be critical for autoinhibition. These results indicate that the head-binding site in the GTD is located in the SD-2. Given the profound interaction between the GTD and the head, it is possible that the GTD undergoes a conformational change upon binding to the head. We thus propose that Mlph-GTBDP binding prevents this conformational change in the GTD, thus inhibiting the interaction between the GTD and the head of Myo5a. These considerations underscore that the detailed structure of Myo5a in its folded conformation remains a central open question.

It is intriguing that the affinity between Mlph-GTBDP and Myo5a-FL (K_d_ of ~20 μM), which is based on the stimulation of Myo5a-FL ATPase activity in this study, was substantially lower than that found between Mlph-GTBDP and isolated GTD (K_d_ of ~0.5 μM)^13^,^27^,^28^. We propose two mechanisms contribute to the low affinity between Mlph-GTBDP and Myo5a-FL. In one mechanism, the binding of the head to the GTD inhibits its interaction with Mlph-GTBDP i.e., the conformation of the Mlph-GTBDP binding site in the GTD is allosterically affected by the head-binding site. As proposed by Luque and Freire^32^, the regulatory and active sites in an allosteric protein must be coupled in such a way that the active site becomes inactive when the regulatory site is occupied and vice versa. Therefore, it is plausible that Mlph-GTBDP binding inhibits the interaction between the GTD and the head, and conversely, head binding inhibits the interaction between the GTD and Mlph-GTBDP.

Alternatively, or in addition, dimerization of GTD might also contribute to the low affinity between Mlph-GTBDP and Myo5a. Thus far, three crystal structures of Myo5a-GTD have been solved^27^,^28^,^33^. In all three structures, Myo5a-GTD forms a head-to-tail dimer (Figure S2A). Although it is not known whether this type of dimerization is an artifact of the crystallization process, it is intriguing that the interface between the two GTDs overlaps the binding site for Mlph-GTBDP (Figure S2B). Moreover, Houdusse and colleagues proposed another type of GTD dimer that is formed via the binding of the N-terminus of one GTD with the Mlph-GTBDP binding site of a symmetry-related GTD^28^. The dimerization of the GTD, regardless of type, likely inhibits the binding of Mlph-GTBDP to the GTD. Our previous work has shown that isolated GTDs are mostly monomers^19^, indicating that the interaction between two isolated GTDs is weak and unlikely to greatly reduce the interaction between the GTD and Mlph-GTBDP. In contrast, the two GTDs in intact Myo5a likely form a dimer, which is stabilized by several segments of coiled-coils preceding the GTD. Therefore, it is possible that the dimerization of the GTDs in intact Myo5a inhibits binding to Mlph-GTBDP, thus reducing the affinity between Mlph-GTBDP and Myo5a. Further experiments are needed to clarify this issue.

In addition to Mlph-GTBDP, Mlph contains another Myo5a-binding site, Mlph-EFBD, which interacts with the exon-F region in the Myo5a tail. Cell biology studies have indicated that for Myo5a to localize to melanosomes in melanocytes, both the interaction between Mlph-EFBD and the exon-F region and the interaction between Mlph-GTBDP and the GTD are essential, but neither is sufficient^14^. Biochemical analysis and yeast two-hybrid experiments have shown that the interaction between Mlph-EFBD and exon-F plays a predominant role in the binding of the two proteins and suggest that the interaction between the GTD and Mlph-GTBD may further stabilize the complex^11^,^25^. Consistently, we found that although Mlph-GTDBP alone is sufficient for fully activating Myo5a motor function *in vitro*, the affinity between Mlph-GTBDP and Myo5a-FL is low (~20 μM). Therefore, we propose following scenario for the formation of the Myo5a/Mlph complex and activation of Myo5a *in vivo*: Mlph first binds to the Myo5a exon-F region, positioning Mlph-GTBDP in close proximity to the GTD. Mlph-GTBDP then binds to the GTD and allosterically inhibits the interaction between the GTD and the head of Myo5a. These two interactions between Mlph and Myo5a stabilize the complex.

The proposed allosteric mechanism by which Mlph-GTBDP regulates the motor function of Myo5a might represent a general mechanism for the regulation of the class V myosin motor function by other cargo-binding proteins. Class V myosin is responsible for transportation of multiple cargoes that attach to the GTD via cargo-binding proteins. It is known that the GTD interacts with different cargo-binding proteins through distinct binding sites, including many that do not overlap with the putative motor domain binding site^29^. Our finding that the GTD is an allosteric protein suggests that other cargo-binding proteins are potentially capable of activating the class V myosin motor function by allosterically inhibiting the interaction between the GTD and the head.

## Materials and Methods

### Proteins

Full-length mouse melanocyte-type myosin-5a (Myo5a-FL) and myosin-5a HMM (Myo5a-HMM, residues 1-1234) were expressed in sf9 cells and prepared as described^21^. The GST-GTD fusion protein, which comprised the C-terminal 410 residues of Myo5a, was expressed in *E. coli* and prepared as described^21^. Point mutations in GST-GTD were introduced with Quikchange site-directed mutagenesis using Ultra High Fidelity Pfu (Stratagene, La Jolla, CA). cDNAs derived from PCR were sequenced to confirm the presence of the intended mutations and the absence of unintended mutations. To remove the GST tag, the GST-GTD protein was bound to GSH-Sepharose and treated with 3–10 U/ml thrombin (Sigma-Aldrich, St.Louis, MO) overnight on ice.

GST-GTBDP (a GST fusion protein containing residues 176–201 of Mlph) was constructed by subcloning the corresponding cDNA of Mlph^25^ into the pGEX4T2 vector using BamH1 and Xho1 sites. GST-GTBDP was expressed in *E. coli* BL21 cells and purified using GSH-Sepharose according to standard procedures. The concentration of GST-GTBDP was measured by taking the absorbance at 280 nm using a molar extinction coefficient of 41160 liters mol^−1^ cm^−1^.

Mlph-GTBDP (>95% pure), which contained a C-terminal tyrosine for accurate concentration determination, was synthesized by Invitrogen Co. (Beijing, China). To remove any possible contaminating small molecules, GTBDP was dissolved in 0.005% trifluoreacetic acid and passed through a disposable column (GE Healthcare) pre-equilibrated with 0.005% trifluoreacetic acid. Fractions containing high concentrations of GTBDP were pooled, and the concentration of GTBDP was determined by measuring the absorbance at 280 nm using a molar extinction coefficient of 1280 liters mol^−1^ cm^−1^.

### GST pull-down

GST pull-down of GST-GTD with Myo5a-HMM was performed as follows: Ten microliters of GSH-Sepharose was added to 100 μl of 0.5 μM GST-GTD and 1 μM Myo5a-HMM in WB-50 buffer (20 mM MOPS-KOH, pH 7.0, 50 mM NaCl, 1 mM MgCl_2_, 1 mM DTT, 12 μM CaM, 1 mM EGTA) and the indicated concentrations of Mlph-GTBDP with rotation at 4 °C for 2 hr. After washing with 200 μl WB-50 buffer three times to remove unbound proteins, bound proteins were eluted twice with 20 μl 10 mM GSH. Eluted proteins were separated by SDS-PAGE (4–20%) and visualized by Coomassie Blue staining. The amount of GST pull-downed interacting proteins was quantified using ImageJ version 1.42Q.

GST pull-down of GST-GTBDP with GTD was performed as described above with minor modifications. GTD was prepared from GST-GTD by thrombin digestion (see above). One hundred microliters of 2 μM GST-GTBDP in WB-150 (20 mM MOPS-KOH, pH 7.0, 150 mM NaCl, 1 mM MgCl_2_, 1 mM DTT, and 1 mM EGTA) was incubated with 20 μl of GSH-Sepharose at 4 °C for 1 hr with rotation. After washing away unbound protein, 100 μl of 4 μM GTD in WB-150 was added to GST-GTBDP-bound GSH-Sepharose and incubated at 4 °C for 30 min with rotation. After washing with 200 μl of WB-100 three times to remove unbound proteins, bound proteins were eluted twice with 20 μl of 10 mM GSH, analyzed by SDS-PAGE (4–20%), and visualized by Coomassie Blue staining.

### Analytical Ultracentrifugation

The sedimentation coefficient of Myo5a-FL was measured by velocity analytical ultracentrifugation at 40,000 rpm at 20 °C as described previously^16^,^21^ with minor modifications. Purified Myo5a-FL was dialyzed against 20 mM MOPS-KOH (pH7.0), 0.15 M KCl, 1 mM MgCl_2_, 1 mM DTT, and 1 mM EGTA on ice overnight. For control sample under EGTA conditions, the dialyzed samples were used directly. For Mlph-GTBDP sample, Mlph-GTBDP was added at a concentration of 40 μM prior to running. For control sample under pCa4 conditions, the CaCl_2_ concentration was adjusted to 1.1 mM prior to running. The density and viscosity for the EGTA conditions calculated with Sednterp were 1.0056 g/ml and 1.00202 cp, respectively. The values for the pCa4 conditions were 1.0057 g/ml and 1.00252 cp, respectively. The partial specific volume of Myo5a-FL was 0.7323. Sedimentation values were determined by curve fitting using the DCDT+ program.

### Negative-Staining Electron Microscopy

The effects of Mlph-GTBDP on the conformation of Myo5a-FL were analyzed by negative-staining electron microscopy. Myo5a-FL was diluted to 100 nM with 150 mM KCl, 10 mM potassium phosphate, pH 7.5, 5 mM MgCl_2_, 1 mM EGTA, and 1 mM DTT on ice. The carbon-coated grids were glow-discharged for 40 sec at middle stage (Harrick Glow-Discharge system) before use to improve the preservation of the Myo5a conformation. Ten microliters of the solution was applied to the glow-discharged grids for 1 min and then stained with 1% uranyl acetate without intermediate rinsing. The same procedure was used for samples of Myo5a-FL containing Mlph-GTBDP with the exception that 100 nM Myo5a was premixed with 40 μM Mlph-GTBDP on ice for approximately 30 min before application to the grids. Negatively stained grids were examined with a Tecnai Spirit100 kV electron microscope (FEI) operated at 120 kV. Micrographs were recorded with an aside-inserted CCD (OSIS MEGAVIEW G2) camera at a magnification of 87,000.

### ATPase Assay

The ATPase activity of Myo5a was measured using an ATP regeneration system at 25 °C as previously described^21^. Unless otherwise indicated, the ATPase activity of Myo5a-FL was measured at 25 °C in 20 mM MOPS-KOH, pH 7.0, 0.2 M KCl, 1 mM MgCl_2_, 1 mM DTT, 0.25 mg/ml BSA, 12 μM CaM, 0.5 mM ATP, 2.5 mM phosphoenolpyruvate, 20 U/ml pyruvate kinase, 1 mM EGTA, ~100 nM Myo5a-FL, and the indicated concentrations for actin and Mlph-GTBDP. The assay under pCa4 conditions was performed similarly with the exception that 1.1 mM CaCl_2_ was added (free Ca^2+^ was approximately 0.1 mM). The ATPase activity of Myo5a-HMM was measured at 25 °C in 20 mM MOPS-KOH, pH 7.0, 0.1 M NaCl, 1 mM MgCl_2_, 1 mM DTT, 0.25 mg/ml BSA, 12 μM CaM, 0.5 mM ATP, 2.5 mM phosphoenolpyruvate, 20 U/ml pyruvate kinase, 1 mM EGTA, ~30 nM Myo5a-HMM, and the indicated concentrations of actin, GST-GTD, and Mlph-GTBDP.

## Additional Information

**How to cite this article**: Yao, L.-L. *et al.* Melanophilin Stimulates Myosin-5a Motor Function by Allosterically Inhibiting the Interaction between the Head and Tail of Myosin-5a. *Sci. Rep.* **5**, 10874; doi: 10.1038/srep10874 (2015).

## Supplementary Material

Supplementary Information

## References

[b1] Berg J. S., Powell B. C. & Cheney R. E. A millennial myosin census. Mol Biol Cell 12, 780–794 (2001). 11294886 10.1091/mbc.12.4.780 PMC32266

[b2] Mehta A. D. *et al.* Myosin-V is a processive actin-based motor. Nature 400, 590–593 (1999). 10448864 10.1038/23072

[b3] Walker M. L. *et al.* Two-headed binding of a processive myosin to F-actin. Nature 405, 804–807 (2000). 10866203 10.1038/35015592

[b4] Tanaka H. *et al.* The motor domain determines the large step of myosin-V. Nature 415, 192–195 (2002). 11805840 10.1038/415192a

[b5] Veigel C., Wang F., Bartoo M. L., Sellers J. R. & Molloy J. E. The gated gait of the processive molecular motor, myosin V. Nat Cell Biol 4, 59–65 (2002). 11740494 10.1038/ncb732

[b6] Sakamoto T. *et al.* Neck length and processivity of myosin V. Journal of Biological Chemistry 278, 29201–29207 (2003). 12740393 10.1074/jbc.M303662200

[b7] Cheney R. E. *et al.* Brain myosin-V is a two-headed unconventional myosin with motor activity. Cell 75, 13–23 (1993). 8402892 10.1016/S0092-8674(05)80080-7

[b8] Hammer J. A. 3rd & Sellers J. R. Walking to work: roles for class V myosins as cargo transporters. Nat Rev Mol Cell Biol 13, 13–26 (2012). 10.1038/nrm3248 22146746

[b9] Wu X. S. *et al.* Identification of an organelle receptor for myosin-Va. Nat Cell Biol 4, 271–278 (2002). 11887186 10.1038/ncb760

[b10] Fukuda M., Kuroda T. S. & Mikoshiba K. Slac2-a/melanophilin, the missing link between Rab27 and myosin Va: implications of a tripartite protein complex for melanosome transport. J Biol Chem 277, 12432–12436 (2002). 11856727 10.1074/jbc.C200005200

[b11] Strom M., Hume A. N., Tarafder A. K., Barkagianni E. & Seabra M. C. A family of Rab27-binding proteins. Melanophilin links Rab27a and myosin Va function in melanosome transport. J Biol Chem 277, 25423–25430 (2002). 11980908 10.1074/jbc.M202574200

[b12] Fukuda M. & Itoh T. Slac2-a/melanophilin contains multiple PEST-like sequences that are highly sensitive to proteolysis. J Biol Chem 279, 22314–22321 (2004). 15145961 10.1074/jbc.401791200

[b13] Geething N. C. & Spudich J. A. Identification of a minimal myosin Va binding site within an intrinsically unstructured domain of melanophilin. J Biol Chem 282, 21518–21528 (2007). 17513864 10.1074/jbc.M701932200

[b14] Wu X., Wang F., Rao K., Sellers J. R. & Hammer J. A. 3rd. Rab27a is an essential component of melanosome receptor for myosin Va. Mol Biol Cell 13, 1735–1749 (2002). 12006666 10.1091/mbc.01-12-0595 PMC111140

[b15] Wang F. *et al.* Regulated conformation of myosin V. J Biol Chem 279, 2333–2336 (2004). 14634000 10.1074/jbc.C300488200

[b16] Li X. D., Mabuchi K., Ikebe R. & Ikebe M. Ca2+-induced activation of ATPase activity of myosin Va is accompanied with a large conformational change. Biochem Biophys Res Commun 315, 538–545 (2004). 14975734 10.1016/j.bbrc.2004.01.084

[b17] Krementsov D. N., Krementsova E. B. & Trybus K. M. Myosin V: regulation by calcium, calmodulin, and the tail domain. J Cell Biol 164, 877–886 (2004). 15007063 10.1083/jcb.200310065 PMC2172279

[b18] Trybus K. M., Krementsova E. & Freyzon Y. Kinetic characterization of a monomeric unconventional myosin V construct. J Biol Chem 274, 27448–27456 (1999). 10488077 10.1074/jbc.274.39.27448

[b19] Li X. D., Jung H. S., Mabuchi K., Craig R. & Ikebe M. The globular tail domain of myosin Va functions as an inhibitor of the myosin Va motor. J Biol Chem 281, 21789–21798 (2006). 16757473 10.1074/jbc.M602957200

[b20] Thirumurugan K., Sakamoto T., Hammer J. A., Sellers J. R. & Knight P. J. The cargo-binding domain regulates structure and activity of myosin 5. Nature 442, 212–215 (2006). 16838021 10.1038/nature04865 PMC1852638

[b21] Li X. D. *et al.* The globular tail domain puts on the brake to stop the ATPase cycle of myosin Va. Proc Natl Acad Sci USA 105, 1140–1145 (2008). 18216256 10.1073/pnas.0709741105 PMC2234105

[b22] Wang F. *et al.* Effect of ADP and ionic strength on the kinetic and motile properties of recombinant mouse myosin V. J Biol Chem 275, 4329–4335 (2000). 10660602 10.1074/jbc.275.6.4329

[b23] Homma K., Saito J., Ikebe R. & Ikebe M. Ca(2+)-dependent regulation of the motor activity of myosin V. J Biol Chem 275, 34766–34771 (2000). 10945977 10.1074/jbc.M003132200

[b24] Lu Z. *et al.* Calmodulin bound to the first IQ motif is responsible for calcium-dependent regulation of myosin 5a. J Biol Chem 287, 16530–16540 (2012). 22437832 10.1074/jbc.M112.343079 PMC3351354

[b25] Li X. D., Ikebe R. & Ikebe M. Activation of myosin Va function by melanophilin, a specific docking partner of myosin Va. J Biol Chem 280, 17815–17822 (2005). 15760894 10.1074/jbc.M413295200

[b26] Sckolnick M., Krementsova E. B., Warshaw D. M. & Trybus K. M. More than just a cargo adapter, melanophilin prolongs and slows processive runs of myosin Va. J Biol Chem 288, 29313–29322 (2013). 23979131 10.1074/jbc.M113.476929 PMC3795233

[b27] Wei Z., Liu X., Yu C. & Zhang M. Structural basis of cargo recognitions for class V myosins. Proc Natl Acad Sci USA 110, 11314–11319 (2013). 23798443 10.1073/pnas.1306768110 PMC3710815

[b28] Pylypenko O. *et al.* Structural basis of myosin V Rab GTPase-dependent cargo recognition. Proc Natl Acad Sci USA 110, 20443–20448 (2013). 24248336 10.1073/pnas.1314329110 PMC3870677

[b29] Pashkova N., Jin Y., Ramaswamy S. & Weisman L. S. Structural basis for myosin V discrimination between distinct cargoes. EMBO Journal 25, 693–700 (2006). 16437158 10.1038/sj.emboj.7600965 PMC1383548

[b30] Velvarska H. & Niessing D. Structural insights into the globular tails of the human type v myosins myo5a, myo5b, and myo5c. PLoS One 8, e82065 (2013). 24339992 10.1371/journal.pone.0082065 PMC3858360

[b31] Gunasekaran K., Ma B. & Nussinov R. Is allostery an intrinsic property of all dynamic proteins? Proteins 57, 433–443 (2004). 15382234 10.1002/prot.20232

[b32] Luque I. & Freire E. Structural stability of binding sites: consequences for binding affinity and allosteric effects. Proteins Suppl 4, 63–71 (2000). 10.1002/1097-0134(2000)41:4+<63::aid-prot60>3.3.co;2-y 11013401

[b33] Nascimento A. F. *et al.* Structural insights into functional overlapping and differentiation among myosin V motors. J Biol Chem 288, 34131–34145 (2013). 24097982 10.1074/jbc.M113.507202 PMC3837155

