# Supplementary material for: Melanophilin Stimulates Myosin-5a Motor Function by Allosterically Inhibiting the Interaction between the Head and Tail of Myosin-5a
Source: Sci Rep. 2015 Jun 3;5:10874. doi: 10.1038/srep10874 (PMC4454200; doi:10.1038/srep10874)
Supplement: Supplementary Information [file srep10874-s1.pdf]

SUPPLEMENTAL INFORMATION

Melanophilin Stimulates Myosin-5a Motor Function by Allosterically Inhibiting the Interaction between the Head and Tail of Myosin-5a

Lin-Lin Yao[a,1], Qing-Juan Cao[a,b,1], Hai-Man Zhang[a,b,1], Jie Zhang[a], Yang Cao[a], and Xiang-dong Li[a,2]

[a]Group of cell motility and muscle contraction, Chinese Academy of Sciences, Beijing 100101, China;

[b]University of Chinese Academy of Sciences, Beijing 100049, China

[1] These authors contributed equally to this work.

[2]To whom correspondence should be addressed: Xiang-dong Li, Institute of Zoology, Chinese Academy of Sciences, Beijing 100101, China. Telephone: 86-10-6480-6015. Email: lixd@ioz.ac.cn

Supplemental Figure 1

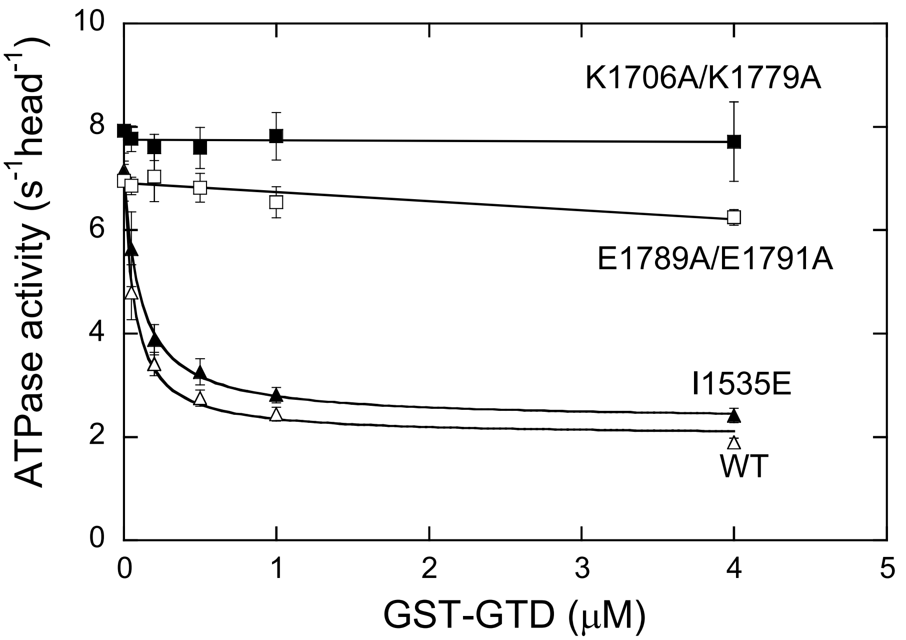

Figure S1. The effects of GTD mutants on the Myo5a-HMM ATPase activity. The Myo5a-HMM ATPase activity was strongly inhibited by GST-GTD-WT and -I1535E mutant. In contrast, the Myo5a-HMM ATPase activity was not inhibited by GST-GTD-K1706A/K1779A mutant and only weakly inhibited by GST-GTD-E1789A/E1791A mutant. The ATPase assays were conducted in 20 mM MOPS-KOH, pH 7.0, 0.1 M NaCl, 1 mM $MgCl_2$, 1 mM DTT, 0.25 mg/ml BSA, 12 μM CaM, 0.5 mM ATP, 2.5 mM phosphoenolpyruvate, 20 U/ml pyruvate kinase, 1 mM EGTA, 40 μM actin, ~30 nM Myo5a-HMM, and 0 - 4 μM GST-GTD. Data are means ± std from three independent assays.

Supplemental Figure 2

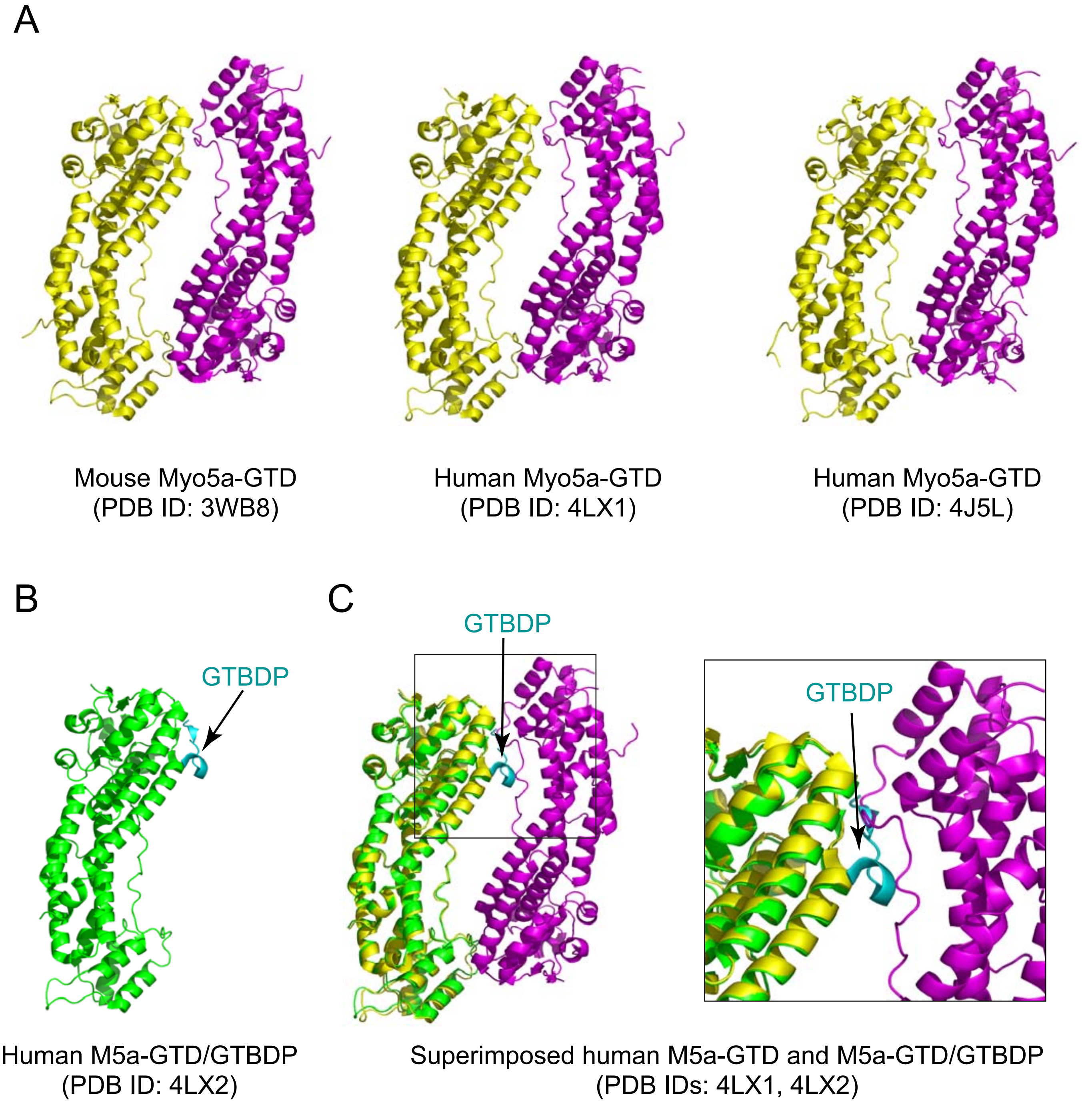

Figure S2. GTBDP binds to the interface of Myo5a-GTD dimer. (A) The crystal structures of human Myo5a-GTD and mouse Myo5a-GTD. There are 4 dimers in the crystal structure of mouse Myo5a-GTD and only one dimer (chain a and c) is shown here. (B) The crystal structure of human Myo5a-GTD/GTBDP complex. (C) Superimposed structures of human Myo5a-GTD (shown in yellow and magenta) and Myo5a-GTD/GTBDP complex (shown in green and cyan).
